# Supplementary material for: Persistence of Low Back Pain and Predictive Ability of Pain Intensity and Disability in Daily Life among Nursery School Workers in Japan: A Five-Year Panel Study
Source: Healthcare (Basel). 2024 Jan 5;12(2):128. doi: 10.3390/healthcare12020128 (PMC10815376; doi:10.3390/healthcare12020128)
Supplement: Supplementary file 1 [file healthcare-12-00128-s001.zip › healthcare-2756588-supplementary.pdf]

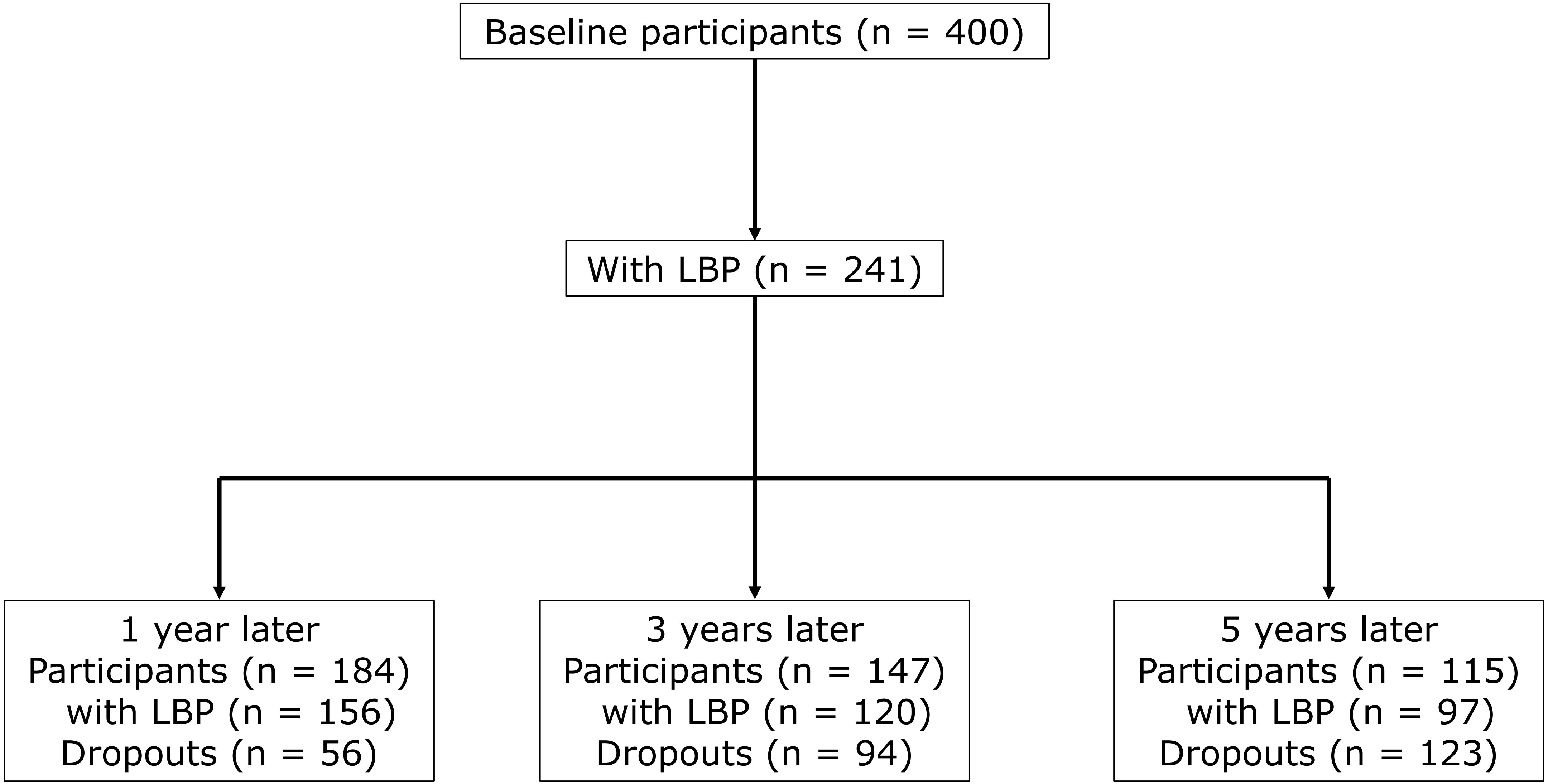

Supplementary Figure S1. Flow of the baseline and follow-up surveys: female subjects.

Supplementary Table S1. Adjusted odds ratios of baseline numerical rating scale (NRS) scores and other characteristics for low back pain (LBP) at 1, 3, and 5 years: logistic regression analyses.

	Adjusted odds ratio (95% confidence interval) for LBP		
	1 year later	3 years later	5 years later
NRS score			
4 or less	1	1	1
5 or greater	4.01 (1.27 – 12.6)*	8.51 (1.87 – 38.7)**	3.49 (0.95 – 12.8)
Sex			
Male	1	1	1
Female	1.28 (0.38 – 4.23)	0.85 (0.22 – 3.33)	0.79 (0.20 – 3.15)
Age (per 1 year increase)	1.01 (0.97 – 1.05)	0.98 (0.95 – 1.02)	1.02 (0.98 – 1.07)
Body mass index			
< 25	1	1	1
≥ 25	4.47 (0.56 – 35.63)	0.59 (0.18 – 1.95)	0.53 (0.14 – 1.98)
Employment status			
Regular	1	1	1
Casual	1.15 (0.29 – 4.56)	1.84 (0.38 – 8.98)	1.20 (0.23 – 6.41)
Occupation			
Cook/nutritionist/other	1	1	1
Teacher	1.49 (0.46 – 4.91)	1.20 (0.32 – 4.53)	0.85 (0.20 – 3.53)
Work schedule			
Regular	1	1	1
Irregular	2.07 (0.70 – 6.14)	1.52 (0.45 – 5.10)	1.23 (0.34 – 4.42)

*: $p < 0.05$; **: $p < 0.01$; ***: $p < 0.001$.

Supplementary Table S2. Adjusted odds ratios of baseline Roland–Morris Disability Questionnaire (RDQ) scores and other characteristics for low back pain (LBP) at 1, 3, and 5 years: logistic regression analyses.

	Adjusted odds ratio (95% confidence interval) for LBP		
	1 year later	3 years later	5 years later
RDQ score			
3 or less	1	1	1
4 or greater	1.92 (0.52 – 7.03)	4.64 (0.98 – 22.0)	7.17 (0.84 – 61.4)
Sex			
Male	1	1	1
Female	1.06 (0.33 – 3.40)	0.61 (0.16 – 2.31)	0.75 (0.19 – 2.96)
Age (per 1 year increase)	1.00 (0.97 – 1.04)	0.98 (0.95 – 1.02)	1.02 (0.97 – 1.06)
Body mass index			
< 25	1	1	1
≥ 25	3.77 (0.48 – 29.44)	0.41 (0.13 – 1.36)	0.41 (0.11 – 1.53)
Employment status			
Regular	1	1	1
Casual	1.23 (0.30 – 4.99)	1.90 (0.38 – 9.41)	0.79 (0.15 – 4.26)
Occupation			
Cook/nutritionist/other	1	1	1
Teacher	1.05 (0.32 – 3.40)	0.87 (0.24 – 3.14)	0.70 (0.17 – 2.90)
Work schedule			
Regular	1	1	1
Irregular	1.80 (0.62 – 5.22)	1.30 (0.39 – 4.31)	1.16 (0.32 – 4.23)

*: $p < 0.05$; **: $p < 0.01$; ***: $p < 0.001$.

Supplementary Table S3. Relationship between the numerical rating scale (NRS) scores at baseline and low back pain (LBP) at 1, 3, and 5 years among only female subjects.

NRS score at baseline	N (%) of those with LBP	Adjusted odds ratio (95% confidence interval) [1)]
1 year later		
4 or less (n = 127)	103 (81.1)	1
5 or greater (n = 52)	49 (94.2)	4.43 (1.20 – 16.5)*
3 years later		
4 or less (n = 99)	84 (75.8)	1
5 or greater (n = 43)	41 (95.3)	6.51 (1.43 – 29.7)*
5 years later		
4 or less (n = 78)	60 (76.9)	1
5 or greater (n = 34)	31 (91.2)	3.06 (0.81 – 11.5)

*: $p < 0.05$.

The NRS scores were unavailable from 5, 5, and 3 participants for the results on LBP 1, 3, and 5 years later, respectively.

1) Sex, age, body mass index, employment status, occupation, and work schedule at the baseline were adjusted.

Supplementary Table S4. Relationship between the Roland–Morris Disability Questionnaire (RDQ) scores at baseline and low back pain (LBP) at 1, 3, and 5 years among only female subjects.

RDQ score at baseline	N (%) of those with LBP	Adjusted odds ratio (95% confidence interval) 1)
1 year later		
3 or less (n = 151)	125 (82.8%)	1
4 or greater (n = 32)	30 (93.8%)	2.56 (0.54 – 12.1)
3 years later		
3 or less (n = 117)	92 (78.6%)	1
4 or greater (n = 29)	27 (93.1%)	4.23 (0.88 – 20.4)
5 years later		
3 or less (n = 92)	72 (78.3%)	1
4 or greater (n = 22)	21 (95.5%)	5.39 (0.64 – 45.4)

*: $p < 0.05$; **: $p < 0.01$.

The RDQ scores were unavailable from 1 participant for the present results.

1) Sex, age, body mass index, employment status, occupation, and work schedule at the baseline were adjusted.
